# Editorial: Affective brain-computer interface in emotion artificial intelligence and medical engineering

**DOI:** 10.3389/fncom.2023.1237252

**Published:** 2023-07-11

**Authors:** Zhaohua Lu, Tingwen Wang, Ruirui Zhang

**Affiliations:** School of Computer Science and Technology, Shandong University of Finance and Economics, Jinan, China

**Keywords:** affective brain-computer interface, emotion artificial intelligence, medical engineering, neuroscience, brain network

## Editorial

Emotion has an important impact on human behavior and mental health in people's daily interpersonal communication and behavior decision-making. Recently, with the rapid development of the brain-computer interface (BCI), the emotional and intelligence demand of human-computer interaction is increasing, and the ability of computers to recognize human emotions has become a research hotspot. Affective intelligence realizes the emotional interaction between humans and computers, making computers more intelligently handle human instructions, and has a wide range of applications in the fields of education, medicine, and criminal investigation. Affective BCI is a branch of emotional intelligence and an important way to achieve the goal of affective intelligence research (Shanechi, 2019; Almarri et al., 2021).

Human emotions are controlled by the brain center; therefore, electroencephalography (EEG) signals can fully and accurately reflect human emotions. An affective BCI extracts brain features related to emotional states based on neurophysiological signals. By recognizing, processing, and responding to users' affection (such as emotions or moods), it can identify users' internal states and adjust them in real time to improve brain-computer interaction (Mowla et al., 2020). The research and development of affective BCIs can not only enhance human-computer interaction, but also have significance in the treatment of various neurological diseases (such as depression) and improving the care of severely disabled people.

To promote basic and applied research, technological innovation, and academic exchanges of affective BCI, this article focuses on various new technologies or new methods related to affective BCI, such as EEG signal acquisition and processing methods, neuroimaging technology, neurophysiological feature extraction, and classification algorithms.

EEG is the most widely used physiological signal for emotional BCIs. Currently, two types of EEG acquisition equipment are commonly used: wet and dry electrodes. Wet electrodes have been widely used in BCI systems because of their improved signal quality; however, wet electrode operation is complicated, time-consuming, labor-intensive, and provides a poor experience for patients. Dry electrodes are easy to operate and wear, provides a comfortable experience for patients, and is suitable for ordinary individuals (van Stigt et al., 2023). In this Research Topic, Zhang et al. designed a multi-instruction steady-state visual evoked potential (SSVEP) speller based on a time-space variable frequency SSVEP stimulation mode using dry electrodes. By combining electrooculography (EOG) and SSVEP signals and using time and space coding, the spelling paradigm not only improves the flexibility of BCI spellers, but also greatly increases the number of output instructions of BCI spelling systems in dry-electrode environments, paving the way for portable and comfortable dry-electrode BCI systems in the future. The brain-controlled switch in the study incorporated EOG signals for arousal stimuli because electrical eye signals are easy to detect. However, the system needs to collect blink data of the previous round of subjects in advance, because different subjects have different blink ranges. Therefore, subsequent work may need to combine more robust algorithms to classify the blinking of different subjects to improve the performance of the system.

A limitation of EEG signals is their low spatial resolution. In contrast, oxygen-based brain imaging technology, such as functional magnetic resonance imaging (fMRI), has better spatial resolution and has been widely used in neuroimaging research, which is an important tool for researchers to explore the mechanisms of brain activity (Coupeau et al., 2022). Parallel computing and deep learning have made great progress. Research shows that feature fusion based on different deep neural networks can improve on-chip representation and inter-chip feature extraction, which is conducive to improving the accuracy of medical image segmentation, while increasing network complexity and computing cost. To solve this problem, Fei et al. proposed a symmetric end-to-end trainable hybrid convolutional neural network (HC-Net) for the small-sample characteristics of MRI data and the properties of thick-layer scanning. HC-Net fully uses the spatial information between adjacent sections of the MRI image sequence and combines the continuous sections of three axes for three-dimensional (3D) convolution, which reduces the number of calculations and improves accuracy. The key to the effectiveness of this method is that the use of 3D convolution to fully extract spatial information between data and the reduction of computational load and parameters through 2D convolution overcomes the problems of 3D convolution overfitting and 2D volume underfitting on small samples. Compared with other serial convolutional neural network models, the training complexity is reduced.

The absence of joyfulness is a central symptom of major depressive disorder and may be more than a single component defect of decreased pleasurable response to reward or positive stimuli or a diminished pursuit of reward (Renner et al., 2019). Rather, it may be a barrier to the transition from motivational imagery to a pleasurable experience; however, the underlying mechanism of this defect is unclear. Currently, little is known about the electrophysiological markers of motivational imagery (such as need or reward). Proverbio and Pischedda measured brain-imagined potentials associated with physiological needs and motivational states in this Research Topic. The experiment collected pictograms of the four macro-demand categories most relevant to the possible use of BCI (one of which included emotional states) and recorded the event-related potentials (ERPs) elicited by these stimuli, focusing on the measurement and statistical analysis of anterior N400 and centroparietal late positive potentials. The results showed that ERPs were smaller and more anteriorly distributed during imagery than perception, but showed some similarity in terms of lateralization, distribution, and category response, thus indicating some overlap in neural processing. As for the affective states, regardless of condition, the N400 was of greater amplitude in response to positive states (such as cheerfulness) than negative ones (such as fear or sadness).

The brain comprises complex structures. By representing brain function as measured by EEG, magnetoencephalography, and fMRI as an abstract network, it can be applied to methods of studying complex systems. Brain network analysis methods have been extensively studied in explaining psychiatric disorders whose neural mechanisms are not well understood (Gonuguntla and Kim, 2020). Threshold selection plays an important role in brain network construction. Guo et al. developed a multi-scale brain network modeling analysis method based on topology data analysis and persistent homology theory, which can coherently quantify various persistent topological features at different scales. In this method, the related algorithm and parameters in the data processing of the model were analyzed and some key problems were investigated, including the construction of nodes and the edge weight matrix and the selection of the filtering threshold in this network. Compared to existing methods, this method extracts the topological features of the brain network more accurately and improves the accuracy of diagnosis and classification. In addition, the experimental results of applying the model to task-based patients with schizophrenia suggest that it can be used as a stable biological reference standard for stability and noise immunity.

From the perspective of artificial intelligence and neurocognitive science, emotional intelligence has been widely studied by academics, but research on emotional BCIs started late. Studies presented in the topic about “*Affective brain-computer interface in emotion artificial intelligence and medical engineering*” provide favorable technical support for the further development of emotional BCIs.

## Author contributions

ZL prepared an original draft of the manuscript. ZL, TW, and RZ critically reviewed and edited the manuscript. All authors have reviewed and approved the final manuscript.
